# Protein Requirements of Pre-Menopausal Female Athletes: Systematic Literature Review

**DOI:** 10.3390/nu12113527

**Published:** 2020-11-16

**Authors:** Drew Mercer, Lilia Convit, Dominique Condo, Amelia J. Carr, D. Lee Hamilton, Gary Slater, Rhiannon M. J. Snipe

**Affiliations:** 1 Centre for Sport Research, School of Exercise and Nutrition Sciences, Deakin University, Burwood 3125, Victoria, Australia; drew.mercer@icloud.com (D.M.); lilia.convitcordova@deakin.edu.au (L.C.); dominique.condo@deakin.edu.au (D.C.); amelia.carr@deakin.edu.au (A.J.C.); 2 Institute for Physical Activity and Nutrition Research, School of Exercise and Nutrition Sciences, Deakin University, Geelong 3216, Victoria, Australia; lee.hamilton@deakin.edu.au; 3 School of Health and Sport Sciences, University of the Sunshine Coast, Maroochydore 4558, Queensland, Australia; gslater@usc.edu.au

**Keywords:** amino acids, muscle protein, exercise, menstrual cycle, contraceptives

## Abstract

This systematic literature review aimed to determine the protein requirements of pre-menopausal (e.g., 18–45 years) female athletes and identify if the menstrual cycle phase and/or hormonal contraceptive use influence protein requirements. Four databases were searched for original research containing pre-menopausal female athletes that ingested protein alongside exercise. The Academy of Nutrition and Dietetics Quality Criteria Checklist was used to determine study quality. Fourteen studies, which included 204 recreationally active or competitive females, met the eligibility criteria for inclusion in this review, and all were assessed as positive quality. The estimated average requirement (EAR) for protein intake of pre-menopausal recreational and/or competitive female athletes is similar for those undertaking aerobic endurance (1.28–1.63 g/kg/day), resistance (1.49 g/kg/day) and intermittent exercise (1.41 g/kg/day) of ~60–90 min duration. The optimal acute protein intake and influence of menstrual cycle phase or hormonal contraceptive use on protein requirements could not be determined. However, pre- and post-exercise protein intakes of 0.32–0.38 g/kg have demonstrated beneficial physiological responses in recreational and competitive female athletes completing resistance and intermittent exercise. The protein requirements outlined in this review can be used for planning and assessing protein intakes of recreational and competitive pre-menopausal female athletes.

## 1. Introduction

Dietary protein supports exercise training adaptations, including the remodelling of protein structures and accretion of lean body mass and strength, and contributing to metabolic pathways during exercise [1,2]. Due to the increased protein turnover associated with exercise, current sports nutrition guidelines for daily protein intake (1.2–2.0 g/kg/day) [3] are higher than national dietary guidelines (~0.6–0.8 g/kg/day) that are aimed at preventing nutrient deficiencies in the general population [4,5,6] rather than optimising exercise training adaptations [1,7]. Unfortunately, these sports nutrition guidelines [3] are limited by methodological issues that have been used to determine protein requirements in athletes [8,9] and are primarily based on research investigating the requirements of male athletes. Research that is primarily conducted in male athletes is then applied to female athletes on the basis of similar resting, post-exercise and post-prandial muscle protein synthesis (MPS) responses, and a lack of evidence suggesting that muscle mass influences post-exercise protein requirements [10,11,12]. This research, however, fails to address differences in sex steroid hormones that affect exercise metabolism [13]. For example, there is compelling evidence that females have higher fat and subsequently lower carbohydrate and protein oxidation than males during fasted aerobic endurance exercise [14,15,16] and that these metabolic differences are likely mediated by oestradiol [13]. These metabolic differences are suggested to result in ~15–25% lower protein requirements for female compared to male endurance athletes [13,17]. It is therefore plausible that sex-based differences in protein requirements may exist across different types of exercise (e.g., resistance, aerobic endurance or intermittent (e.g., team sports)), although this is an area that requires further research.

In addition to the type of exercise, hormonal fluctuations across the menstrual cycle may influence resting metabolic rate (e.g., ~9% increase in the luteal phase of the menstrual cycle) [18,19] and the subsequent nutrition and protein requirements of female athletes [20]. The menstrual cycle facilitates female reproduction between menarche and menopause (defined as “pre-menopause” in this review) and can be divided into various phases based on the concentrations of sex steroid hormones [21,22]. Of particular interest is that these fluctuating reproductive hormones coincide with the age of peak competitive performance (e.g., 20–39 years) in female athletes [23]. Moreover, approximately half of pre-menopausal female athletes use hormonal contraceptives (e.g., oral contraceptive pills (OCPs), injections, intrauterine devices, etc.), [24,25] that provide exogenous hormones (e.g., oestrogen and progestin) of varying types and doses that downregulate endogenous hormone production [26]. Recent reviews suggest that these alterations in hormones across different phases of the menstrual cycle and with the use of hormonal contraceptives may have small variable effects on exercise performance, although findings are limited by poor methodological approaches to classifying the phase of menstrual cycle [26,27]. Nevertheless, it is important that future research considers the potential nutrient, exercise and hormone interactions to enable the development of female-specific sports nutrition guidelines.

There are currently no studies that specifically address the protein requirements of female athletes across the menstrual cycle or with the use of hormonal contraceptives. However, there is some evidence that protein catabolism is higher at rest and following aerobic endurance exercise in the luteal phase, when oestrogen and progesterone are elevated, compared to the early follicular phase when oestrogen and progesterone concentrations are low [14,28,29]. In contrast, a more recent study suggests there is no difference in myofibrillar and collagen protein synthesis between the follicular and the luteal phases 24 h after 60 min of resistance exercise [30]. It is therefore currently unclear if these physiological responses to exercise across the menstrual cycle influence the protein requirements of female athletes and if this differs based on the type (e.g., aerobic endurance vs. resistance) of exercise performed. Similarly, it is unclear if the lower post-exercise myofibrillar protein synthesis observed in female athletes using hormonal contraceptives, compared to non-users [31], can be mediated by protein intake.

Considering that adequate dietary protein is important for supporting physiological adaptations to exercise, there is a growing need to determine the protein requirements for pre-menopausal athletes that address the influence of endogenous and exogenous hormones and potential metabolic interactions with different types of exercise. Therefore, this systematic literature review aimed to outline the current evidence on daily and acute (e.g., intake pre-, during or post-exercise) protein requirements of pre-menopausal female athletes undertaking resistance, endurance and intermittent exercise. A secondary aim of this systematic literature review was to identify if the phase of the menstrual cycle and/or hormonal contraceptive use influences the protein requirements of female athletes. The information from this review can be used to inform the planning and assessment of protein intakes for pre-menopausal female athletes and inform the development of future female-specific sports nutrition guidelines.

## 2. Materials and Methods

This systematic literature review was conducted in accordance with the PRISMA statement [32].

### 2.1. Search Strategy

A literature search was conducted in Medline, Embase, Sportdiscus and CINAHL databases from inception to March 2020. Search terms used included a combination of female or woman or contraceptive and “muscle protein synthesis” or “myofibrillar protein” or “protein balance” or “protein and catabolism” or “protein metabolism” or “protein turnover” or “protein and nitrogen balance”. Additional articles were identified by hand-searching the reference lists of review articles and included full-text articles.

### 2.2. Inclusion and Exclusion Criteria

Screening of title and abstracts and full-text articles against inclusion and exclusion criteria was conducted independently by two authors. Disagreements in the screening process were resolved by discussion and consensus. The inclusion criteria consisted of (1) original full-text peer-reviewed research, (2) healthy pre-menopausal or hormonal contraceptive using recreationally, competitive or elite female athletes aged 18–45 years, (3) consumption of daily or acute dose of protein in conjunction with an exercise protocol and (4) determination of protein requirements (e.g., an estimated average requirement (EAR)) and/or a physiological response (e.g., muscle protein synthesis, nitrogen balance, muscular strength, body composition, etc.) to the exercise and protein intake, as per previous reviews and current guidelines on protein intake [2,3,7]. Articles were excluded if they (1) did not report pre-menopausal female data separately from males or post-menopausal females, (2) included sedentary/physically inactive participants, (3) administered amino acids or combinations of amino acids in isolation and/or in conjunction with protein intake or (4) were not published in English.

### 2.3. Data Extraction and Management

Data were extracted into a customised table by one author and reviewed by a second author. For studies that contained both male and female participants, only the female data were retrieved and reported. Studies were divided into aerobic endurance exercise, resistance exercise and intermittent exercise (e.g., intermittent-type activity similar to team sport) based on the exercise performed during the study and the established potential differences in protein requirements based on the different physiological adaptations that exist for these types of exercise, based on existing literature [33,34]. Studies were then divided into daily and acute (e.g., pre-, during or post-exercise dose) protein intake based on the intervention and primary outcome measure to allow for comparison to current sports nutrition guidelines. Protein intakes that were reported in absolute dose (e.g., grams) were converted to relative protein intake (e.g., grams per kilogram of body mass) using the mean body mass of participants for standardisation and comparison to other articles and sports nutrition guidelines. Where articles reported an EAR for protein, this was used to calculate a recommended dietary intake (RDI) (e.g., referred to as recommended dietary allowance (RDA) by the Institute of Medicine [6] and reference nutrient intake by the Department of Health [4]) using the equation: RDI = 1.24 × EAR [5], where 1.24 equates to a 12% coefficient of variation for protein requirements [35]. This method has previously been used to determine the RDI (or equivalent RDA) for the nutrient reference values and dietary reference intakes of healthy individuals [5,6] and was selected due to the large standard deviations and small sample sizes of the studies included in this review.

### 2.4. Quality Assessment

Study quality was assessed independently by two authors using the Academy of Nutrition and Dietetics Quality Criteria Checklist [36] and disagreements in ratings were resolved by discussion and consensus. This checklist consists of ten questions that address scientific validity, including the risk of bias, and provides an overall study rating of negative, neutral or positive [36].

## 3. Results

### 3.1. Screening and Study Selection

**Figure 1:**
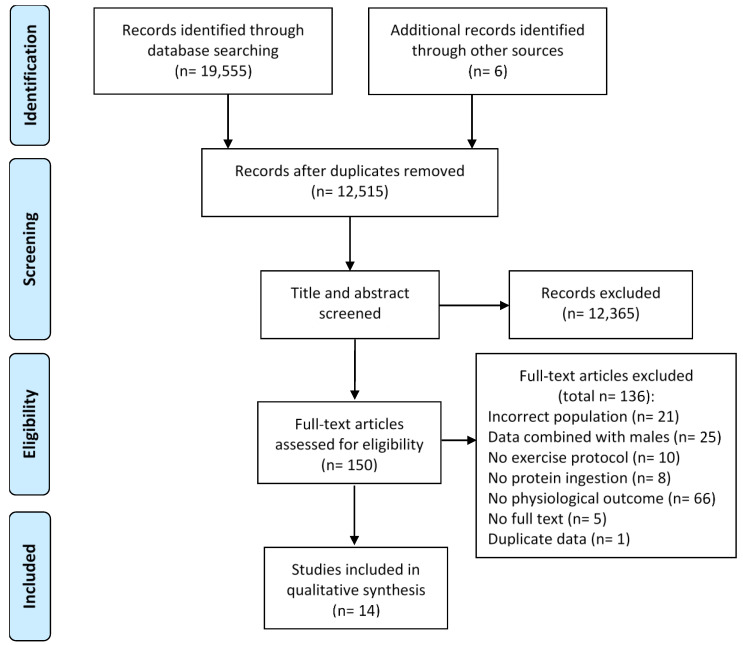
Search and screening flow diagram.

The database search resulted in 19,555 articles, with 12,515 titles and abstracts screened after the removal of duplicates (Figure 1). One hundred and fifty full-text articles were screened and 14 of these articles (studies) met the eligibility criteria and were included in this review (Figure 1).

### 3.2. Quality Assessment of Included Studies

All 14 studies included in this review received a positive overall rating after assessment against the Academy of Nutrition and Dietetics Quality Criteria Checklist [36] (Table 1).

Academy of Nutrition and Dietetics Quality Criteria Checklist [36] for the 14 included studies: Q1, clear research question; Q2, participant selection free from bias; Q3, comparability of study groups; Q4, description of withdrawals; Q5, blinding; Q6, description of study procedures; Q7, outcomes clearly defined and measurements valid and reliable; Q8, appropriate statistical analysis; Q9, results support conclusion; Q10, unlikely bias from funding or sponsorship. For a positive overall rating, the majority of Q1–10 must be met as well as criteria for Q2, Q3, Q6 and Q7. A negative overall rating is provided when the majority of criteria are not met. A neutral rating is provided when criteria for Q2, Q3, Q6 and Q7 are met but not the majority.

### 3.3. Participant Characteristics, Menstrual Cycle and Hormonal Contraceptive Use

A total of 204 recreationally active and/or competitive pre-menopausal female athletes participated in the 14 studies included in this review (Table 2, Table 3 and Table 4). Phase of the menstrual cycle was reported by eight studies, with four of these studies combining pre-menopausal female athletes and female athletes using hormonal contraceptives (Table 2, Table 3 and Table 4). All four studies with female athletes completing aerobic endurance exercise were conducted in the mid-follicular phase of the menstrual cycle and/or combined with hormonal contraceptives users (Table 2). In contrast, two studies with resistance (Table 3) and two with intermittent (Table 4) exercise were performed in the luteal phase and/or combined with hormonal contraceptives. It was not possible to determine the effect of the menstrual cycle and hormonal contraceptive use on the protein requirements of female athletes due to a lack of comparisons available both within and between studies.

### 3.4. Aerobic Endurance Exercise

The daily protein requirements of recreationally active and competitive female endurance athletes were investigated by three studies using the nitrogen balance method (Table 2). An EAR of 1.28 g protein/kg/day (calculated RDI 1.59 g/kg/day) was reported for competitive female cyclists who undertook a four-day exercise protocol that included 150 min of cycling intervals, a repeat sprint cycle test on days two and four with a rest day on day three [42]. In contrast, other studies in this review reported daily protein intakes of 0.8 and 1.4 g/kg/day and resulted in negative nitrogen balance when recreationally active females completed a 90 min run at 65% VO_2max_ and competitive female cyclists completed 90 min cycling intervals each day for three days [16,39]. The latter study in competitive cyclists determined an EAR of 1.63 g protein/kg/day, equating to an RDI of 2.02 g protein/kg/day to achieve nitrogen balance [39].

Acute protein intakes of female athletes completing aerobic endurance exercise were investigated by one study that showed an attenuation in loss of body mass and a trend for improved nitrogen balance after a 7-day exercise protocol when a mixed supplement containing 0.24 g/kg whey protein was consumed post-exercise compared to 10 h pre-exercise [43].

### 3.5. Resistance Exercise

Daily protein intakes of female athletes completing resistance exercise was investigated by three studies (Table 3). Using the indicator of amino acid oxidation (IAAO) technique, one of these studies reported an EAR of 1.49 g protein/kg/day (calculated RDI 1.85 g/kg/day) for recreationally active resistance-trained female athletes completing a single whole-body resistance training session [40]. Findings from an eight-week resistance training study suggest that daily protein intakes of 0.9 g/kg/day and 2.5 g/kg/day are similarly effective at supporting increases in maximal strength, with no significant differences observed between these different protein intakes [38]. However, the increase in fat-free mass (FFM) was greater in female physique athletes that consumed 2.5 g protein/kg/day compared to 0.9 g protein/kg/day [38]. In addition, a study by Tinsley et al. [45] reported that time-restricted feeding to a period of eight hours with protein intakes of 1.6 g/kg/day during eight weeks’ resistance training does not adversely affect maximal strength, endurance or FFM.

Two studies showed that acute protein intakes of ~0.37 g/kg consumed post-resistance exercise support an increased MPS [10], with a similar protein intake (~0.38 g/kg) ingested either pre- or post-resistance exercise supporting increased maximal upper-body strength in recreationally active females [41].

### 3.6. Intermittent Exercise

The daily protein requirements of competitive female athletes completing intermittent exercise (Loughborough intermittent shuttle test) determined an EAR of 1.41 g protein/kg/day (calculated RDI 1.75 g/kg/day, Table 4) using the IAAO method [48]. Acute post-intermittent exercise protein intakes of 0.32–0.38 g/kg during eight weeks of training exerted beneficial physiological effects in four studies, including an attenuation in the decline of reactive strength index post-exercise, reduced fat mass and creatine kinase levels at 24 h post-exercise and increased maximal strength and lean mass [37,44,46,47]. Additionally, a comparison of whey vs. casein protein showed that the type of protein consumed post-exercise did not affect measures of maximal strength or body composition [46].

## 4. Discussion

This systematic literature review aimed to determine the daily and acute protein requirements for pre-menopausal female athletes and identify if the menstrual cycle phase and hormonal contraceptive use influenced these requirements. Very limited research has been conducted on the protein requirements of female athletes, but the quality of research included in this review is positive. A key finding of this review is that the EAR for protein intake of pre-menopausal recreationally active and/or competitive female athletes is similar for aerobic endurance exercise (1.28–1.63 g/kg/day), resistance exercise (1.49 g/kg/day) and intermittent exercise (1.41 g/kg/day). These requirements are within the mid-range of current sports nutrition guidelines (1.2–2.0 g/kg/day) for all athletes [3]. The optimal acute protein dose for female athletes remains to be determined. However, protein intakes of 0.32–0.38 g/kg consumed pre- or post-exercise have demonstrated beneficial physiological responses with resistance and intermittent exercise. Insufficient data were available to determine the impact of menstrual cycle phase and hormonal contraceptive use on the protein requirements of female athletes and is an area deserving further research. To the authors’ knowledge, this is the first systematic literature review to determine the protein requirements of pre-menopausal female athletes. The findings of this review can be used to inform the development of future female-specific sports nutrition guidelines that can be used by sports nutrition professionals to plan and/or assess the adequacy of dietary protein intakes when seeking to optimise physiological responses of pre-menopausal female athletes. This review also highlights a number of key areas for future research.

### 4.1. Aerobic Endurance Exercise

Findings from this review indicate that the EAR for protein intake of competitive female endurance athletes is 1.28–1.63 g/kg/day when completing multiple days of moderate to high-intensity cycling intervals and/or sprints with an exercise duration up to 150 min [39,42]. It should be noted that protein intakes at the EAR will achieve nitrogen balance in ~50% of competitive female cyclists completing an equivalent exercise load and can be used for planning and assessing dietary intakes of groups of female endurance athletes [4,5,6]. However, the calculated RDI of 1.59–2.02 g/kg/day is more appropriate when planning and assessing the daily protein intake of individual athletes and will achieve nitrogen balance in ~97–98% of pre-menopausal female endurance athletes during equivalent exercise [4,5,6]. The requirements (e.g., EAR and RDI) determined from this review, whilst based on limited research, can be used to inform the updating or development of future sports nutrition guidelines. For example, the EAR from this review is within the lower mid-range, whilst the RDI is within the upper mid-range of protein guidelines for all athletes (1.2–2.0 g/kg/day) [3]. The EAR and RDI are, however, higher than previous recommendations for female endurance athletes (0.88–0.94 g/kg/day for moderate intensity and 1.28–1.36 g/kg/day for elite endurance athletes) that were believed to be ~15–25% lower than male athletes [13,17]. Interestingly, the protein requirements based on nitrogen balance studies in this review are similar to those determined by the IAAO method in male endurance athletes (EAR 1.65 g/kg/day and RDI 1.83 g/kg/day) completing a 20 km treadmill run [49].

Differences in protein requirements of male and female endurance athletes are believed to primarily arise from lower protein (and endogenous carbohydrate) oxidation and higher fat oxidation during exercise [14,15,16]. However, these differences in substrate oxidation are diminished with high carbohydrate availability (e.g., high availability of endogenous and exogenous carbohydrates) [50], highlighting the importance for consideration of carbohydrate availability and energy balance on the protein requirements of female endurance athletes. For example, female endurance athletes with low carbohydrate and/or energy availability will likely have increased daily and acute protein requirements due to increased protein oxidation during exercise, and to attenuate loss of lean body mass during intensified training [17,43]. This point is especially prudent considering that inadequate energy intake is a common feature amongst female endurance athletes [51,52]. Furthermore, the protein requirements of female athletes may be increased with higher exercise intensity and longer exercise duration and be influenced by the training status of the athlete [8,17,53,54]. Our findings therefore need to be interpreted and applied with consideration to nutritional status, exercise load and training status of female endurance athletes. Moreover, it is possible that protein requirements of female endurance athletes in this review have been overestimated due to high daily protein intakes and the limitations of the nitrogen balance method for estimating protein requirements of athletes, see the review by Tipton and Witard [8]. Recent research has also indicated that the IAAO method can overestimate protein requirements in athletes with high habitual protein intakes and requires ≥5 days of dietary adaptation, which is not commonly employed by studies using this method [9]. Further research using sound methodological design is therefore required to enhance our current knowledge on protein requirements and the practical application of daily protein intake recommendations for pre-menopausal female endurance athletes across a range of contexts, such as menstrual cycle phase, energy balance status and training modality.

The optimal acute protein dose for female endurance athletes could not be determined from the one study included in this review that provided a set protein dose as part of a mixed macronutrient supplement at 10 h pre- or post-exercise [43]. While research on the optimal acute protein dose in female endurance athletes is lacking, a recent dose–response investigation in male endurance athletes showed a post-exercise protein dose of 0.49 g/kg is required to maximally stimulate myofibrillar protein synthesis, and a ~0.58 g/kg dose optimised whole-body protein balance and de novo mitochondrial and myofibrillar protein synthesis [55]. Interestingly, these requirements are higher than post-resistance exercise protein requirements (0.31 g/kg for myofibrillar protein synthesis) reported in a recent review [11], suggesting endurance athletes should place a greater emphasis on protein intake post-exercise [56]. While the optimal acute protein dose remains to be determined, the studies in this review demonstrate that post-exercise protein intake is likely to be beneficial and should be considered an important strategy when planning dietary protein intakes of pre-menopausal female endurance athletes.

### 4.2. Resistance Exercise

Current evidence from this review shows that the EAR and RDI for protein intake of recreationally active female athletes completing a single whole-body resistance training session is 1.49 g/kg/day and 1.85 g/kg/day, respectively [40]. However, longer-term (e.g., eight weeks) resistance training studies consisting of two to four training sessions per week suggest that daily protein intakes ranging from 0.9–2.5 g/kg/day can support increases in maximal strength in recreationally trained and physique sport female athletes [38,45]. The beneficial response across a large protein intake may be attributed to the exercise training stimulus contributing to maximal strength responses to a greater extent than daily protein intakes, which have only been shown to augment strength by ~9% [2]. In contrast to maximal strength, higher protein intakes (1.6 g/kg/day) may be required to support increases in FFM during long-term resistance training in female athletes [38,45].

The daily protein requirements of resistance-trained females in this review are, with the exception of maximal strength, within the mid- to upper range of protein recommendations for all athletes (1.2–2.0 g/kg/day) [3] and are similar to a recent meta-analysis that suggested an EAR of 1.62 g/kg/day for resistance-trained male and female athletes [2]. It is important to note that several factors may affect these daily protein requirements, including training load, habitual protein intake and training status. For example, highly trained athletes may have reduced requirements due to lower protein turnover, however, higher habitual protein intake and/or training load may offset and/or increase protein requirements [57,58]. Furthermore, the daily protein requirements of female resistance-trained athletes are likely to be higher during energy restriction for preventing muscle mass loss [59]. However, such assumptions are based on male athletes, and further research is required to determine if training volume, intensity, fitness level, habitual protein intake and energy balance impact the daily protein requirements of resistance-trained female athletes to the same extent as male athletes.

Protein dose–response studies aimed at determining the amount of protein required for inducing maximal post-resistance exercise MPS have exclusively been performed in recreationally active males [60,61,62]. Whist some research suggests a similar post-resistance exercise MPS between males and females and in response to protein feeding [10,30,63], we do not have definitive evidence of the dose required to achieve maximal MPS in females. Two studies in this review have observed beneficial responses, such as increased MPS and upper-body strength, in recreationally active females with protein doses of ~0.37–0.38 g/kg consumed pre- and post-resistance exercise [10,41]. However, these intakes may exceed the requirements for optimal post-exercise MPS, which have been reported as 0.31 g/kg protein, based on research that was predominantly conducted in males [60]. Considering excessive intakes may contribute to increased amino acid oxidation and displacement of other important nutrients (e.g., carbohydrate and/or energy intake) [11,64], future research should be conducted to determine the protein dose required to achieve maximal post-resistance exercise MPS in pre-menopausal female athletes.

### 4.3. Intermittent Exercise

Findings from one study in this review suggest the EAR and RDI for protein are 1.41 g/kg/day and 1.75 g/kg/day, respectively, for competitive female sport athletes completing 60 min of intermittent variable-intensity exercise [48]. These protein requirements are within the mid- (EAR) to upper (RDI) range of general protein recommendations for all athletes (1.2–2.0 g/kg/day) [3]. In addition, the protein requirements are similar to the aforementioned requirements of endurance and resistance-trained athletes. It should be noted, however, that these requirements are based on limited research and that the protein requirements of female athletes completing intermittent exercise are likely to vary considerably depending on the physiological requirements of the exercise performed (e.g., exercise mode, intensity, duration, rest periods, exercise order, etc.) [65]. Further research is therefore required to determine how these factors influence the protein requirements of female athletes completing various types of intermittent (e.g., sport-specific) exercise.

Acute post-intermittent exercise and/or pre- and post-intermittent exercise protein doses in the range of 0.32–0.39 g/kg were shown to have beneficial physiological responses (increased maximal strength, improved body composition and post-exercise recovery) in competitive female basketballers, dancers and resistance-trained females [37,44,46,47]. Interestingly, the average daily protein intakes (1.1–1.39 g/kg/day) in two of these studies [44,47] were below the daily protein EAR from this review (1.41 g/kg/day). Improvements in maximal strength and agility in these studies may therefore be attributed to enhanced recovery [37] from the post-exercise protein intake and/or the training stimulus exerting a greater ergogenic response than the protein intake [2]. Similar to endurance and resistance exercise, the optimal acute protein dose cannot be determined but is likely below 0.32–0.39 g/kg, and is an area deserving further research.

### 4.4. Influence of Menstrual Cycle and Hormonal Contraceptives on the Protein Requirements of Pre-Menopausal Female Athletes

The effect of hormone variations across the menstrual cycle and with hormonal contraceptive use on the protein requirements of female athletes completing endurance, resistance and intermittent exercise could not be determined from this review due to insufficient research exploring and/or controlling for these factors. Despite this lack of research, it is plausible that the protein (and/or specific amino acid) requirements of female athletes may be increased during the luteal phase of the menstrual cycle when progesterone and oestrogen levels are both elevated. A decrease in plasma amino acids during the luteal, compared to the follicular, phase of the menstrual cycle has been reported by several studies in healthy non-athletic females [66,67,68,69,70,71] and may be attributed to increased cell cycle progression and growth, and endometrial protein biosynthesis [69,72]. Increased nitrogen utilisation and excretion in healthy females [29], and increased protein catabolism and decreased plasma amino acids in female endurance athletes, have also been observed during the luteal phase of the menstrual cycle [14,73], but the physiological implications of these responses on protein requirements remain to be explored.

In contrast, there is some evidence to suggest that protein requirements may not vary across the menstrual cycle. A cross-sectional study of female athletes in the follicular (*n* = 8) and in the luteal (*n* = 7) phases of the menstrual cycle showed no difference in muscle myofibrillar or collagen protein fractional synthetic rate (FSR) at 24 h post-endurance exercise [30]. It is, however, unknown if the lack of difference in this study is attributed to the cross-sectional design, phase of the menstrual cycle measured (e.g., early vs. mid-luteal phase), exercise protocol, protein intake and/or outcome measures studied. Menstrual cycle disturbances, such as amenorrhea, anovulation and luteal phase deficiency, are common in female athletes and may also contribute to variability in research findings if the phase and hormonal fluctuations of a normal menstrual cycle are not appropriately verified [74]. Future research with the appropriate methodological design is therefore required to determine if the protein requirements of female athletes are altered due to changes in hormone concentrations across the menstrual cycle.

Similarly, further research is required to determine the effect of hormonal contraceptives on the protein requirements of female athletes. The blood amino acid profile of contraceptive users has consistently been shown to differ from non-contraceptive users [75,76,77], although it is currently unknown if this alteration in amino acid metabolism affects protein requirements at rest or in response to exercise. Any potential alteration in protein (and/or amino acid) requirements of female athletes using hormonal contraceptives may also depend on the type and/or dose of hormones administered [75]. For example, a study has shown that female athletes using third-generation (i.e., low androgenic activity progestins) OCPs had impaired myofibrillar protein FSR at rest and 24 h post-endurance exercise compared to females using second-generation OCPs or in the follicular phase of the menstrual cycle [31]. Subsequent research has, however, shown that OCP users had an increase in type I muscle fibre cross-sectional area and a trend for increased muscle mass compared to non-OCP users during 10 weeks’ resistance training [78]. Interestingly, the OCP group had significantly higher protein intakes than the non-OCP users (1.3 ± 0.2 g/kg/day vs. 1.1 ± 0.2 g/kg/day), although it is unknown if differences in protein intake contributed to these results [78]. Considering the wide variety of hormonal contraceptives available and that usage in female athletes is very common [24,25], future research into the impact of hormonal contraceptive use on the protein requirements of female athletes is warranted.

### 4.5. Protein Type, Timing and Distribution

It should be noted that most of the studies in this review have used high biological value (e.g., high digestibility, absorption and essential amino acid content) protein sources, such as whey or simulated egg-based protein, that maximise post-exercise MPS in male athletes when compared to plant-based sources [79,80,81,82]. Considering female athletes have demonstrated similar post-exercise and protein feeding MPS responses to male athletes [10], it is possible that higher protein intakes than outlined in this review may be required when lower biological value (e.g., plant-based) proteins are consumed [82,83]. This may be of particular concern to female athletes who are more likely than male athletes to follow specific diets, including vegetarian and vegan diets, that contain lower biological value proteins compared to omnivorous diets [84,85].

Research from this review supports the notion that early (i.e., within 1 h) post-exercise protein intake is beneficial for female athletes, potentially due to enhanced availability of amino acids for protein anabolism [86,87]. Protein intake consumed immediately post-exercise improved nitrogen balance and maintenance of body mass in female endurance athletes compared to protein intake consumed 10 h pre-exercise [43]. Additionally, protein consumed either 15 min prior to or post-exercise has been shown to support increases in maximal upper body strength in females during resistance training [41]. Protein timing, however, may not be as important as the amount of protein consumed, which has shown to contribute to muscle hypertrophy to a greater extent than timing in male and female athletes [88,89]. Moreover, emerging research on pre-sleep protein intake in pre-menopausal female athletes has shown no and/or trivial effects on next-morning endurance and resistance training exercise performance [90,91]. Further research is therefore required to determine the importance of protein timing on the physiological adaptations of pre-menopausal female athletes.

The distribution of protein intake across the day can also influence protein anabolism with optimised post-exercise intake (~0.25 g/kg) every three hours, promoting greater myofibrillar protein synthesis than smaller doses (~0.12 g/kg) consumed more frequently (every 1.5 h) or larger doses (~0.49 g/kg) consumed less frequently (every 6 h) [86]. A recent study in male athletes has also demonstrated that evenly distributed protein intake across three meals augments muscle hypertrophy with resistance training to a greater extent than uneven protein distribution across meals [92]. The optimal distribution of protein intake has not been studied in female athletes, but sports nutrition guidelines recommend protein doses should be consumed every three to four hours across the day [7]. These recommendations, however, are in contrast to a study within this review that showed time-restricted feeding to a period of eight hours had no adverse effect on maximal strength and body composition compared to equivalent protein intake (0.39 g/kg post-exercise dose and daily intake of 1.6 g/kg/day) with unrestricted feeding in female resistance-trained athletes [45]. Whilst the distribution of protein intake cannot be determined from this study, the eight-hour feeding window would not allow the aforementioned “optimal” distribution to be achieved. The conflicting findings between current recommendations based on acute post-exercise responses and longer-term training studies may be, at least partially, attributed to the adequate daily and acute post-exercise protein intakes, maximised number of feeding occasions and/or co-ingestion of other nutrients in the training studies [45,93].

While this review has focused on protein requirements, the translation and practical application of these requirements into sports nutrition guidelines need to be considered alongside female athletes’ requirements for other nutrients, such as carbohydrate, which have clear links to exercise performance outcomes [3]. Despite the requirements and guidelines being provided based on body mass, it may be challenging for female athletes to achieve high protein intakes alongside the requirement for other nutrients due to generally lower overall energy requirements and intakes compared to male athletes [94,95,96]. Studies in this review have reported the contribution of daily energy intake from protein in the range of 19–41% is beneficial in supporting physiological responses, whereas intakes at 15–16% of total energy were inadequate. Considering that protein intake is positively correlated with energy intake in athletes [97], achieving adequate total daily energy intake may be an important strategy that supports optimal protein intakes in female athletes. Research on dietary intakes of female athletes suggests endurance (mean intake 1.5 ± 0.4 g/kg/day) and resistance-trained (mean intake 1.5 ± 0.5 g/kg/day) athletes achieve the EARs from this review [97]. However, the protein intake of female intermittent athletes (mean intake 1.3 ± 0.3 g/kg/day) were below the EAR of 1.41 g/kg/day [97] but would be deemed adequate if compared to the lower end of current guidelines (1.2 g/kg/day) [3]. Considering that the EAR represents a 50% probability of adequate intake, it is possible that many female athletes may be at risk of inadequate protein intake, although further research is required to determine this.

A strength of this review is the novel focus on female athletes and that the findings of this review are based on positive quality studies, suggesting a low risk of bias. However, our findings are limited by the small number of studies with recreationally active or competitive female athletes performing up to four days per week of resistance or intermittent exercise and 60–180 min of endurance exercise. The findings of this review therefore do not necessarily reflect the exercise schedule, intensity, duration, fitness levels and energy balance of elite female athletes and the implications of these factors on their protein requirements. This review has highlighted several significant gaps in the current research, of which the biggest omission is the lack of research investigating the impact of endogenous and exogenous female sex steroid hormones on the protein requirements of pre-menopausal female athletes. Determining the impact of both endogenous and exogenous hormones on the protein requirements of female athletes will be important for the future use of female athletes in sports science research, and also have practical outcomes for the development of sports nutrition guidelines aimed at optimising exercise training adaptation and post-exercise recovery.

## 5. Conclusions

The EAR and RDI for protein intake of pre-menopausal recreational and/or competitive female athletes are within the mid- to upper range of current sports nutrition guidelines (1.2–2.0 g/kg/day); and are similar for aerobic endurance (EAR 1.28–1.63 g/kg/day, RDI 1.59–2.02 g/kg/day), resistance (EAR 1.49 g/kg/day, RDI 1.85 g/kg/day) and intermittent exercise (EAR 1.41 g/kg/day, RDI 1.75 g/kg/day). These protein requirements can be used as a starting point for planning or assessing daily protein intakes of recreational and competitive female athletes completing ~60–90 min of exercise. The optimal acute (i.e., pre-, during or post-exercise) protein dose for female athletes remains to be determined. However, pre- and post-exercise protein intakes of 0.32–0.38 g/kg have demonstrated beneficial physiological responses in recreational and competitive female athletes completing resistance and intermittent exercise. The influence of menstrual cycle phase and hormonal contraceptive use on these protein requirements is unclear and requires further research. This review provides a foundation for female-specific protein requirements and the future development of sports nutrition guidelines that address the unique physiology of females and aim to enhance the preparation, performance and recovery of female athletes.

## Figures and Tables

**Table 1:** Quality assessment of included studies.

Author and Year	Q1	Q2	Q3	Q4	Q5	Q6	Q7	Q8	Q9	Q10	Overall Rating
Brown et al., 2018 [37]	Y	Y	Y	N/A	Y	Y	Y	Y	Y	Y	Positive
Campbell et al., 2018 [38]	Y	Y	Y	Y	N	Y	Y	Y	Y	N	Positive
Houltham and Rowlands 2014 [39]	Y	Y	Y	N/A	N	Y	Y	Y	Y	Y	Positive
Malowany et al., 2019 [40]	Y	Y	Y	N	N	Y	Y	Y	Y	Y	Positive
Phillips et al., 1993 [16]	Y	Y	N/A	N/A	N	Y	Y	Y	Y	Y	Positive
Pihoker et al., 2019 [41]	Y	Y	Y	Y	N	Y	Y	Y	Y	Y	Positive
Rowlands and Wadsworth 2011 [42]	Y	Y	Y	N	Y	Y	Y	Y	Y	Y	Positive
Roy et al., 2002 [43]	Y	Y	Y	N	Y	Y	Y	Y	Y	U	Positive
Taylor et al., 2016 [44]	Y	Y	Y	N	Y	Y	Y	Y	Y	Y	Positive
Tinsley et al., 2019 [45]	Y	Y	Y	Y	Y	Y	Y	Y	Y	Y	Positive
West et al., 2012 [10]	Y	Y	N/A	N/A	N	Y	Y	Y	Y	Y	Positive
Wilborn et al., 2013 [46]	Y	Y	Y	N	Y	Y	Y	Y	N	U	Positive
Wilborn et al., 2016 [47]	Y	Y	N/A	N	Y	Y	Y	Y	Y	Y	Positive
Wooding et al., 2017 [48]	Y	Y	Y	N	N	Y	Y	Y	Y	Y	Positive

Y, yes (criteria met); N, no (criteria not met); U, unclear; N/A, not applicable.

**Table 2:** Protein requirements of female athletes completing aerobic endurance exercise.

Author/Year (Study Design)	Female Athletes	Menstrual Cycle/Contraceptives	Exercise Protocol	Protein Intake	Control/Comparison Intake	Outcome(s) from Protein Intake ^1^
Daily Protein Requirements						
Houltham and Rowlands 2014 [39] (cross-over)	10 competitive cyclists and triathletes 61.3 ± 5.4 kg Body fat % NR	Mid-follicular (day 4–11)	90 min cycle intervals at 50–70% VO_2max_ for 3 days	Daily protein intake 2.7 g/kg/day (includes mean 0.75 g/kg whey protein post-exercise) (daily energy: 32% protein, 45% CHO, 23% fat)	Daily protein intake 1.4 g/kg/day (habitual) (daily energy: 16% protein, 54% CHO, 30% fat)	Positive nitrogen balance vs. negative nitrogen balance in control EAR 1.63 g/kg/day RDI ^2^ 2.02 g/kg/day
Phillips et al. 1993 [16] (single intervention) ^3^	Six recreationally active students 58.1 ± 5.4 kg Body fat 18.8 ± 1.7%	Mid-follicular (day 4–11)	90 min run at 65% VO_2max_	Daily protein intake 0.8 g/kg/day (breakfast energy: 4% protein, 82% CHO, 14% fat)	N/A	Negative nitrogen balance
Rowlands and Wadsworth 2011 [42] (cross-over)	12 competitive cyclists 60.8 ± 3.4 kg Body fat 19 ± 3%	Six mid-follicular (day 3–7), six hormonal contraceptives	150 min cycle intervals at 50–90% *W*_max_ day 1, sprint performance test (10 × workload max sprints) days 2 and 4	Protein blend 0.7 g/kg/h (with 1.4 g/kg/h CHO and 0.26 g/kg/h fat; energy 30% protein, 59% CHO, 11% fat) for 4 h post-exercise with high daily carbohydrate diet	Isocaloric control, protein 0.1 g/kg/h (with 2.1 g/kg/h CHO and 0.26 g/kg/h fat; energy 4% protein, 85% CHO, 11% fat) for 4 h post-exercise with high daily carbohydrate diet	Positive nitrogen balance vs. negative nitrogen balance in control EAR 1.28 g/kg/day RDI ^2^ 1.59 g/kg/day
Acute Protein Requirements						
Roy et al. 2002 [43] (cross-over)	10 recreationally trained endurance athletes 61.6 ± 7.6 kg Body fat 21.9 ± 1.1%	Four mid-follicular (day 4–11), six triphasic OCP	60 min cycle at 65% VO_2peak_ on days 1, 3, 4 and for 90 min day 6, plus cycle to exhaustion (75% VO_2peak_) on day 7	Post-exercise: mixed supplement 0.24 g/kg whey protein (energy 23% protein, 66% CHO, 12% fat) non-caloric placebo 10 h pre-exercise (daily energy: 16% protein, 58% CHO, 26% fat)	Pre-exercise: mixed supplement 0.24 g/kg whey protein 10 h pre-exercise (energy 23% protein, 66% CHO, 12% fat) non-caloric placebo post-exercise (daily energy: 16% protein, 58% CHO, 26% fat)	No differences in nitrogen balance (trend for improved balance on days 6 and 7 with post-exercise) ↓ body mass loss vs. pre-exercise

EAR, estimated average requirement; CHO, carbohydrate; N/A, not applicable; NR, not reported; OCP, oral contraceptive pill; RDI, recommended dietary intake; VO_2max_, maximal oxygen uptake; VO_2peak_, peak oxygen uptake; W_max_, watts maximum. ^1^ Differences refer to statistical significance reported in the study. ^2^ RDI calculated as 12% coefficient of variation (1.24 × EAR) in accordance with Rand et al. [35]. ^3^ Comparison group data did not meet the inclusion criteria.

**Table 3:** Protein requirements of female athletes completing resistance exercise.

Author/Year (Study Design)	Female Athletes	Menstrual Cycle/Contraceptives	Exercise Protocol	Protein Intake	Control/Comparison Intake	Outcome(s) from Protein Intake ^1^
Daily Protein Requirements						
Malowany et al. 2019 [40] (cross-over)	Eight recreationally active RT athletes 67.0 ± 7.7 kg Body fat 24.4 ± 6.9%	Luteal (days NR)	Single whole-body RT session	Isocaloric meal with 0.2–2.9 g/kg/day crystalline amino acid based on egg protein provided in eight hourly doses post-exercise (% energy NR)	N/A	EAR 1.49 g/kg/day RDI ^2^ 1.85 g/kg/day Nitrogen balance 1.53 g/kg/day
Campbell et al. 2018 [38] (cohort study)	17 physique athletes (*n* = 8 intervention, *n* = 9 control) 61.0 ± 6.1 kg Body fat 22.7 ± 3.0%	NR	Eight-week whole-body RT program, two to four sessions/week	Daily protein intake 2.5 g/kg/day (includes mean 0.41 g/kg whey protein pre- and post-exercise. Daily energy: 41% protein, 41% CHO, 18% fat)	Daily protein intake 0.9 g/kg/day (includes acute mean 0.08 g/kg pre- and post-exercise. Daily energy: 19% protein, 62% CHO, 19% fat)	↑ maximal strength in both groups ↑ FFM higher vs. control
Tinsley et al. 2019 [45] (cohort study)	17 recreationally active RT athletes (*n* = 9 intervention, *n* = 8 control) 63.9 ± 7.8 kg Body fat < 33%	NR	Eight-week whole-body RT program, three sessions/week	Daily protein intake 1.6 g/kg/day (includes mean 0.39 g/kg whey protein post-exercise. Daily energy: 27% protein, 42% CHO, 34% fat)	Time-restricted (8 h) feeding with daily protein intake 1.6 g/kg/day (includes mean 0.39 g/kg whey protein post-exercise. Daily energy: 27% protein, 39% CHO, 32% fat)	↑ maximal strength, endurance and FFM in both groups
Acute Protein Requirements						
West et al. 2012. [10] (single intervention) ^3^	Eight recreationally active 67.1 ± 5.6 kg Body fat 23.1 ± 4.1%	Four pre-menopausal (phase NR), four OCP	Single lower-body RT session	0.37 g/kg whey protein post-exercise (daily energy: 15% protein, 55% CHO, 30% fat)	N/A	↑ MPS early (1–5 h) and late (24–28 h) post-exercise
Pihoker et al. 2019 [41] (cohort study)	43 recreationally active (*n* = 17 pre-exercise and *n* = 17 post-exercise, *n* = 9 control) 66.5 ± 11.4 kg Body fat % NR	NR	Six-week whole-body RT program, two sessions/week	Pre-exercise group: mixed supplement 0.38 g/kg whey and casein protein Post-exercise group: mixed supplement 0.38 g/kg whey and casein protein (supplement energy: 56% protein, 36% CHO, 8% fat)	No nutrition intake	↑ maximal upper body strength vs. control No difference in lower body strength or body composition between groups

EAR, estimated average requirement; CHO, carbohydrate; FFM, fat-free mass; whole-body, includes upper and lower body exercises; MPS, myofibrillar protein synthesis; N/A, not applicable; NR, not reported; OCP, oral contraceptive pill; RDI, recommended dietary intake; RT, resistance training. ^1^ Differences refer to statistical significance reported in the study. ^2^ RDI calculated as 12% coefficient of variation (1.24 × EAR) in accordance with Rand et al. [35]. ^3^ Comparison group data did not meet the inclusion criteria. ↑, increase; ↓, decrease.

**Table 4:** Protein requirements of female athletes completing intermittent exercise.

Author/Year (Study Design)	Female Athletes	Menstrual Cycle/Contraceptives	Exercise Protocol	Protein Dose, Type, Timing	Control/Comparison Intake	Outcome(s) from Protein Intake ^1^
Daily Protein Requirements						
Wooding et al. 2017 [48] (cross-over)	Six competitive rowing, ice hockey, volleyball athletes 68.8 ± 4.1 kg Body fat 21.8 ± 2.7%	Luteal (days NR)	Modified Loughborough test (4 × 15 min variable intensity shuttle run)	Isocaloric meal with 0.2–2.66 g/kg/day crystalline amino acids based on egg protein provided in eight hourly doses post-exercise (% energy NR)	N/A	EAR 1.41 g/kg/day RDI ^2^ 1.75 g/kg/day
Acute Protein Requirements						
Brown et al. 2018 [37] (cohort)	20 competitive dancers (*n* = 10 intervention, *n* = 10 control) 61.8 ± 7.9 kg Body fat % NR	Six luteal, 14 hormonal contraceptives (groups NR)	15 × 30 m repeated sprints	0.32 g/kg whey protein immediately and 2 h post-exercise (energy 91% protein, 8% CHO, 1% fat) (average daily protein intake of 1.8 g/kg/day. Daily energy: 21% protein, 63% CHO, 24% fat)	0.32 g/kg carbohydrate immediately and 2 h post-exercise (energy 0% protein, 99.5% CHO, 0.5% fat) (daily protein intake 1.3 g/kg/day. Daily energy: 15% protein, 61% CHO, 25% fat)	↓ decline in reactive strength index during 72 h post-exercise ↓ CK levels at 24 h post-exercise
Wilborn et al. 2016 [47] (single intervention) ^3^	Nine resistance-trained athletes 65.1 ± 8.4 kg Body fat 25.5 ± 7.2%	NR	Eight-week whole-body intermittent exercise program, four sessions/week	0.38 g/kg whey protein post-exercise (energy 96% protein, 4% CHO, 0% fat) (average daily protein intake 1.1 g/kg/day)	N/A	↑ maximal strength and agility
Taylor et al. 2016 [44] (cohort)	14 competitive basketballers (*n* = 8 intervention 66.0 ± 3.1 kg, body fat 25.4 ± 4.2%; *n* = 6 control 68.2 ± 7.6 kg, body fat 25.1 ± 4.7%)	NR	Eight-week whole body anaerobic, agility and RT program, four sessions/week	0.36 g/kg whey protein pre- and post-exercise (energy 96% protein, 0% CHO, 4% fat) (average daily protein intake 1.39 g/kg/day)	0.35 g/kg maltodextrin pre- and post-exercise (energy 0% protein, 100% CHO, 0% fat) (daily protein intake of 1.08 g/kg/day)	↑ maximal strength and agility scores vs. control
Wilborn et al. 2013 [46] (cohort)	16 competitive basketballers (*n* = 8 intervention 66.0 ± 4.9 kg, body fat 27.0 ± 4.9%; *n* = 8 comparison 68.0 ± 2.9 kg, body fat 25.0 ± 5.7%)	NR	Eight-week whole body anaerobic, agility and RT program, four sessions/week	0.36 g/kg whey protein pre- and post-exercise (energy 83% protein, 14% CHO, 3% fat) (daily protein intake NR)	0.35 g/kg casein protein pre- and post-exercise (energy 86% protein, 11% CHO, 4% fat) (daily protein intake NR)	↑ maximal strength, lean mass and anaerobic performance, and ↓ in fat mass in both groups

CK, creatine kinase; EAR, estimated average requirement; CHO, carbohydrate; whole body, includes upper and lower body; N/A, not applicable; NR, not reported; RDI, recommended dietary intake; RT, resistance training. ^1^ Differences refer to statistical significance reported in the study. ^2^ RDI calculated as 12% coefficient of variation (1.24 × EAR) in accordance with Rand et al. [35]. ^3^ Comparison group data did not meet the inclusion criteria.
